# Pamphlets Received

**Published:** 1876-08

**Authors:** 


PAMPHLETS RECEIVED.—On Spasmodic Urethral Stricture. By F. M. Otis, M.D., Professor of Genito-Urinary Diseases at the College of Physicians and Surgeons, New York. Urethrotomy; External and Internal combined, in case of multiple and difficult stricture, with remarks on Urethral Calibre; A case of Reflex Neuralgia, associated with Urethral Contraction and a rare form of Urinary Sinus, with a description of the cold water coil. By the same author.

A Surgical study: Gastrotomy and Gastrostomy. By J. H. Pooley, M.D., Professor of Surgery, Starling Medical College, Columbus, Ohio.

Address of J. H. Van Deman, M.D., President. Read before the Medical Society of Tennessee, at Chattanooga, Hamilton county, April, 1876.

On the Use of Baths in Typhoid Fever. By J. G. Thomas, M.D., Savannah, Ga.

Second Annual Report of the Board of Health of the city of Mobile, for the year 1875.

On the Use of Sulphate of Cinchonidia in parts of the States of Illinois, Indiana, Missouri, Kentucky, and the Mississippi Valley, in 1875, from medical journals, societies and individual physicians.

Proceedings of the Medical Society of the county of Kings, Brooklyn, N. Y. Medical and scientific papers, reports, discussions, and notes, published monthly, in the interest of the profession of Kings county; for June, 1876.

Proceedings of the State Medical Society, held at Ann Harbor, Michigan, May 10th, 11th, and 12th, 1876.

Transactions of the State Medical Society of Arkansas, 1875–6.

Transactions of the Medical Society of the District of Columbia, April, 1876.

Sixth Annual Report of the New York Ophthalmic and Aural Institute, 46 East Twelfth street, for the year beginning January 1st, 1875, and ending December 31st, 1875. Dr. H. Knapp, surgeon.

Sixteenth Annual Announcement of the Miami Medical College, of Cincinnati, Ohio. Session of 1876–7.

Annual Circular of the Medical Department of the University of Louisiana, for the session of 1876–7, and catalogue of session of 1875–6.

Fifty-second Annual Announcement of the Jefferson Medical College, of Philadelphia, for the session of 1876–7, beginning on Monday, October 2d.

